# Retraction: Prevalence of coronary artery disease and its risk factors in Majmaah City, Kingdom of Saudi Arabia

**DOI:** 10.3389/fcvm.2023.1355005

**Published:** 2023-12-29

**Authors:** Frontiers Editorial Office

A Retraction of the Original Research Article Prevalence of coronary artery disease and its risk factors in Majmaah City, Kingdom of Saudi Arabia By Albar HM, Alahmdi RA, Almedimigh AA, Shaik RA, Ahmad MS, Almutairi AB, Alghuyaythat WKZ, Alaoufi SY, Aloyari WO and Alanazi RMS (2022). Front. Cardiovasc. Med. 9:943611. doi: 10.3389/fcvm.2022.943611

The journal retracts the 2022 article cited above.

Following publication, concerns were raised regarding the contributions of the authors of the article. Our investigation, conducted in accordance with Frontiers policies, confirmed a serious breach of our authorship policies and of publication ethics; the article is therefore retracted.

This retraction was approved by the Chief Executive Editor of Frontiers. The authors do not agree with this retraction.

